# Influence of Manufacturing Parameters and Post Processing on the Electrical Conductivity of Extrusion-Based 3D Printed Nanocomposite Parts

**DOI:** 10.3390/polym12040733

**Published:** 2020-03-25

**Authors:** Rubén Paz, Rocío Moriche, Mario Monzón, Joshua García

**Affiliations:** 1 Departamento de Ingeniería Mecánica, Universidad de Las Palmas de Gran Canaria, 35017 Las Palmas de Gran Canaria, Spain; mario.monzon@ulpgc.es (M.M.); joshua.garcia@ulpgc.es (J.G.); 2 Departamento de Física de la Materia Condensada, ICMS, CSIC-Universidad de Sevilla, Apartado 1065, 41080 Sevilla, Spain

**Keywords:** extrusion-based AM, 3D printing, graphene nanoplatelets, ABS, manufacturing parameters, post processing, neosanding, volume and surface electrical conductivity

## Abstract

The influence of manufacturing parameters of filament extrusion and extrusion-based Additive Manufacturing (AM), as well as different post processing techniques, on the electrical conductivity of 3D printed parts of graphene nanoplatelets (GNP)-reinforced acrylonitrile butadiene styrene (ABS) has been analyzed. The key role of the manufacturing parameters to obtain electrically conductive filaments and 3D printed parts has been demonstrated. Results have shown that an increase in extrusion speed, as well as lower land lengths, induces higher extrudate swelling, with the consequent reduction of the electrical conductivity. Additionally, filaments with lower diameter values, which result in a higher surface-to-cross-section ratio, have considerably lower electrical conductivities. These factors tune the values of the volume and surface electrical conductivity between 10^−4^–10^0^ S/m and 10^−8^–10^−3^ S/sq, respectively. The volume and surface electrical conductivity considerably diminished after 3D printing. They increased when using higher printing layer thickness and width and were ranging between 10^−7^–10^−4^ S/m and 10^−8^–10^−5^ S/sq, respectively. This is attributed to the higher cross section area of the individual printed lines. The effect of different post processing (acetone vapor polishing, plasma and neosanding, which is a novel finishing process) on 3D printed parts in morphology and surface electrical conductivity was also analyzed.

## 1. Introduction

During recent decades, electrically conductive polymer composite materials have attracted an increasing interest as they are potential candidates to be used in a wide range of industries such as automotive, aeronautical and renewable energy [1,2,3]. Several authors have analyzed the electrical conductivity of different composite materials, such as ternary composite films [4], carbon black or carbon fiber-filled poly(methyl methacrylate) nanocomposites [5,6], or even triple hierarchic poly (ethylene terephthalate), carbon black and thermoplastic polyurethane composites for strain sensing applications [7].

Particularly, carbon-based nanoreinforcement, such as carbon nanotubes (CNTs) [8,9] and graphene nanoplatelets (GNPs) [10,11], has been widely investigated. Percolation thresholds for GNPs-based nanocomposites have been published to be between 1 to 10 wt %, depending on the lateral dimensions of the nanoplatelets and their thickness [12], as well as the dispersion degree and orientation [13].

Nowadays, Additive Manufacturing (AM) is an emerging field as it brings the possibility of building parts with complex geometries, which are difficult to obtain by conventional methods [14]. The main advantages of this new technology is the shortening of manufacturing cycles and the reduction of production costs [15], which increase its competitiveness compared with other methods [16]. Extrusion-based AM (ISO/ASTM 52900:2015), commonly known as Fused Deposition Modelling (FDM), consists in the deposition of melted thermoplastic filaments sequentially, layer by layer, through a nozzle tip [17]. In this technology, the addition of nanoreinforcement into the polymeric matrix causes a significant drop of the melt flow index (MFI) due to an increase in viscosity [18]. Additionally, forces applied during extrusion through a capillary, i.e., extrusion die, and shear rates during the process may cause modifications on the distribution and orientation of the nanoreinforcement and polymeric chains [19], leading to changes in electrical properties, which can give rise to loss of electrical conductivity in printed parts. This phenomenon has been described by Dorigato et al. [18], who reported that the electrical conductivity of resultant 3D-printed parts is strongly dependent on the 3D printing direction and lower than that of the filament. Although these differences in electrical conductivity have been reported, deeper research to generate knowledge and understanding of mechanisms taking place needs to be assessed.

Some authors [20] have developed electrically conductive filament composite for AM by mixing polyetheretherketone (PEEK), carbon nanotubes and graphite nanoplates, obtaining an electrical conductivity in the range of 1.5–13.1 S/m. The inclusion of graphite nanoplates reduced the coefficient of friction by up to 60%, keeping the electrical conductivity. Other authors [21] formulated a filament composite of graphite/polylactic acid (PLA) for AM, applied to lithium-ion batteries, adding and optimizing plasticizers to provide the required flexibility of the filament in the 3D printer.

Furthermore, due to the technological importance of ABS in industry, different surface post processing methods have been used to modify surface properties in order to enhance finishing or activate the surface to metalize it [22]. Two of the more common processing methods are vapor polishing [23] and plasma [24]. Currently, there is an emerging post processing named neosanding, which is implemented in 3D printers and can be applied while the 3D parts are being built. This technique (also called “ironing” in some slicer software) consists in repeating the final layer without extruding material, at a higher feed rate and keeping the high temperature of the nozzle. This allows the removal of ridges, resulting in a smoother surface. All these post processing methods induces surface modifications that can also modify the electrical conductivity of the 3D printed parts.

This work analyses the influence of manufacturing parameters of filament extrusion and extrusion-based AM, as well as different post processing techniques, on the electrical conductivity of 3D printed parts of GNP-reinforced acrylonitrile butadiene styrene (ABS). Changes induced in dispersion and orientation of the GNPs, which determine the electrical performance of 3D printed parts, along the transformation from the pellet to the final part are discussed.

## 2. Materials and Methods

### 2.1. Materials

Pellets of GNP/ABS nanocomposite with a GNP content of 15 wt %, which is above the percolation threshold, were acquired, supplied by Centro Tecnológico Fundación AITIIP (Zaragoza, Spain). The ABS grade was ABS Magnum^TM^ 3452 (from Styron company, Berwyn, PA, USA). Table 1 shows its properties.

**Figure 1:**
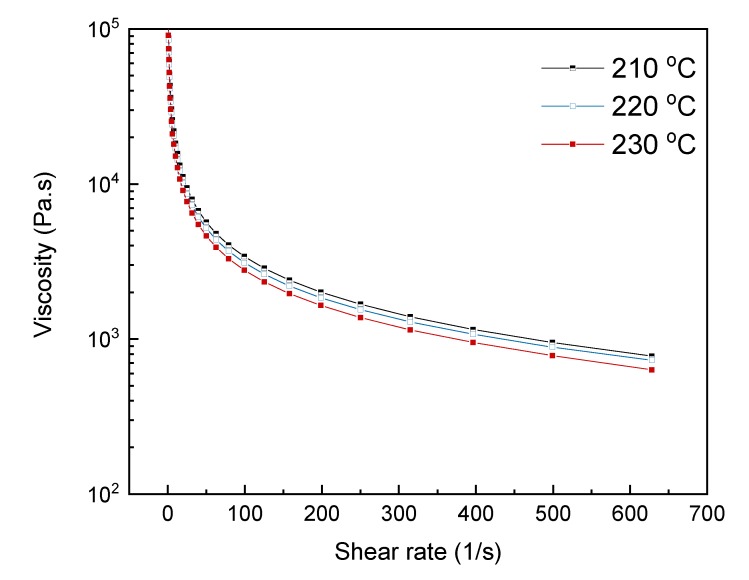
Viscosity curves of the composite material at 210, 220 and 230 °C.

A rheology analysis of the composite material was carried out in an AR-G2 Magnetic Bearing Rheometer (TA Instruments, New Castle, DE, USA). The analysis consisted in a frequency sweep from 100 to 0.1 Hz at three temperatures (210, 220 and 230 °C). Figure 1 shows the three curves obtained. The viscosity was lower at higher temperatures, but without significant differences.

### 2.2. Manufacturing

#### 2.2.1. Filament

Extrusion process was used to obtain the GNP/ABS nanocomposite filament from the original pellets. The extrusion was carried out in a Noztek Touch extruder (Noztek, Shoreham-by-Sea, UK) using a temperature of 195–220 °C. Two screw speeds were used to analyze the influence on the electrical conductivity of the filaments. Three different refrigeration configurations were used for the filament manufacturing: air cooling (without fan, NF), linear fan (LF) and annular fan (circular air flow around the filament, AF). Additionally, different extrusion dies were used, one with a conical output (provided with the extrusion machine, with 0.93° output angle and 16.63 mm) and another one with cylindrical output (M, CNC machined with 1.763 mm diameter and 18.50 mm land length). These two morphologies were used to analyze the influence of the land length of the extrusion die in the properties of the filaments. Table 2 shows the process conditions for the produced filaments and the code for each filament configuration.

#### 2.2.2. 3D printed Parts

The filaments extruded that showed the highest electrical conductivity were used to print 3D parts. An FDM Prusa i3 3D printer (Prusa Research, Prague, Czech Republic) was used. Parts were printed with a nozzle temperature of 230 °C. Despite the optimal extrusion temperature for the filament production was 220 °C, in this case the nozzle tip diameter is significantly lower (0.4 mm) compared with the extrusion die diameter (1.7 mm), reason why a slightly higher temperature was used. Indeed, according to the literature [25] and filament manufacturers, the recommended extrusion temperature for ABS and ABS composites is between 230–240 °C. Regarding the deposition speed, values around 40 mm/s were found in the literature for ABS composite 3D printing [26]. However, preliminary 3D printing tests were made at 35 mm/s and the result was not successful. The high viscosity of the material led to a lower flow, thus depositing less material than needed and causing the stretching and subsequent dragging of the filament. For this reason, the speed was gradually reduced until achieving a good deposition, which occurred at 20 mm/s. The samples were 3D printed with dimensions of 10 × 10 × 10 mm^3^. Different layer thicknesses and extrusion widths were used with the aim of analyzing their influence on the electrical conductivity of printed parts. A rectilinear infill with 100% density and three perimeters were applied. Table 3 shows the details of the operational parameters.

#### 2.2.3. Post Processing

Three different prost processing methods were applied to the surface of 3D-printed parts with the aim of enhancing the surface electrical conductivity. Post processing was carried out in parts printed with the operational parameters that led to the highest electrical conductivity.

- Acetone post processing. Vapor polishing of 3D-printed parts was carried out under acetone vapor generated at 57 °C for 1 min in a closed content. After surface post processing, samples were dried at 37 °C.
- Plasma post processing. Plasma surface post-processing was conducted in a Zepto plasma unit (Electronic Diener, Plasma Surface Technology). The plasma was generated in O_2_ atmosphere with a pressure of 1.8 mbar and a power of 70 W [24]. Surfaces of 3D-printed parts were exposed to the oxygen plasma for 3 min.
- Neosanding. Neosanding is a novel in-situ surface post processing that was applied with a 90° path to the direction of the deposited filament of the last layer. The nozzle tip temperature was maintained and the height of the path was reduced 0.05 mm compared with the height of the last printed layer (0.05 mm penetration). On the other hand, the extrusion width was reduced to 0.05 mm. This low value was used as a strategy to avoid the filament extrusion and achieve the neosanding post processing, since the slicer software (Simplify3D) does not include this option. The effect of this process on the electrical conductivity was assessed.

### 2.3. Morphological and Microstructural Characterization

Analysis of microstructural features and morphology of 3D-printed parts were also analyzed by optical microscopy Olympus BX51 (Olympus Corporation, Tokyo, Japan).

Roughness and surface profiles of printed and post processed 3D parts were obtained by using a Mitutoyo SJ-201P roughness tester following the ISO 4287-1997 standard.

### 2.4. Measurement of Volume and Surface Electrical Conductivity

#### 2.4.1. Volume Electrical Conductivity

**Figure 2:**
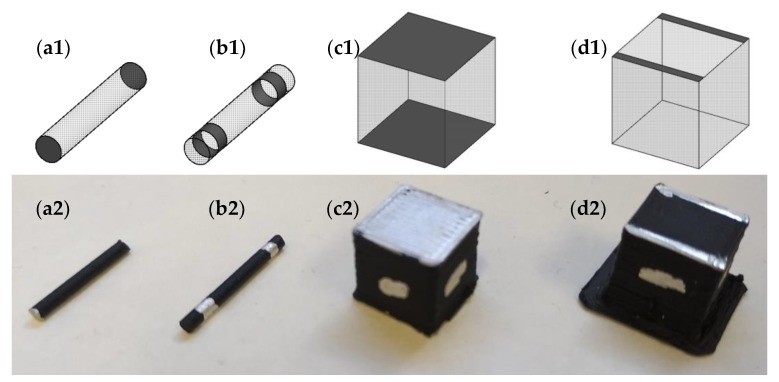
Samples for measurement of (**a**,**c**) volume and (**b**,**d**) surface electrical conductivity of: (**a**,**b**) filaments and (**c**,**d**) 3D printed parts (1 index: schematic representation; 2 index: real part).

The volume electrical conductivity ($\sigma_{v}$) of the pellets, filaments and 3D-printed parts were measured by using the ASTM D257 standard method. In the case of the pellets and filaments, two opposite cross-sections of cylinders with a length of ~10 mm were painted with silver paint in order to minimize the electrical contact resistance (Figure 2(a1,a2)). In the case of 3D-printed parts, two opposite cross-sections with dimensions of 10 × 10 mm^2^ were painted with silver (Figure 2(c1,c2)) and the electrical resistance was measured along three directions, i.e., *X*, *Y* and *Z* printing axis, with a Keithley 2400 Source Meter (Mitutoyo Corporation, Kawasaki, Japan). The volume electrical conductivity was calculated from the electrical resistance following Equation (1).

(1) $$\sigma_{v}=\frac{1}{R}\cdot \frac{l}{A_{c}},$$

where R is the measured electrical resistance, l is the length between electrical contacts and $A_{c}$ is the electrical contact area.

#### 2.4.2. Surface Electrical Conductivity

The surface electrical conductivity, per square, ($\sigma_{s}$) of the pellets, filaments and 3D-printed parts were measured by using the ASTM D257 standard method. Two silver paint rings were used in pellets and filaments sections to measure the electrical resistance in order to minimize the electrical contact resistance (Figure 2(b1,b2)). In the case of 3D-printed parts, two lines of silver paint were used as contact (Figure 2(d1,d2)). The distance between the electrodes in all the cases was ~10 mm. The surface electrical conductivity (per square) was calculated from the electrical resistance, which was measured with a Keithley 2400 Source Meter (Keithley Instruments, Cleveland, OH, USA), following Equation (2).

(2) $$\sigma_{v}=\frac{1}{R}\cdot \frac{l}{P_{c}},$$

where R is the measured electrical resistance, l is the length between electrical contacts and $P_{c}$ is the electrical contact perimeter.

## 3. Results

### 3.1. Measurement of Volume and Surface Electrical Conductivity

**Figure 3:**
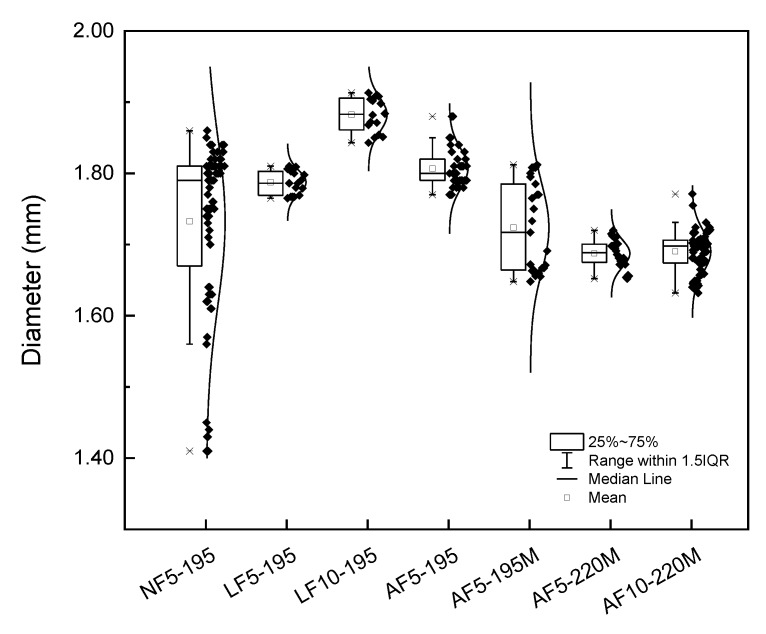
Influence of operational parameters on filament diameter distribution (asterisks represent the outliers, black squares the data and the curved lines the density functions).

Optimization of the filament extrusion process is a key factor to ensure the quality in printed parts. In order to feed the printing extrusion tip, a uniform diameter of 1.7 mm along the filament is needed. Figure 3 shows normal distributions of the diameter of filaments obtained with the extrusion dies with the conical and cylindrical output geometry, specified in Table 2. For the conical output geometry, when no fan was used to refrigerate the filament (NF5-195), a wide distribution was obtained with diameters in the range of 1.55–1.87 mm. In contrast, with a fan cooling system, the normal distribution became narrower and thus the diameter along the filament was more uniform. In this case, differences obtained in diameter normal distribution between filaments extruded by using linear fan (LF5-195) and annular fan (AF5-195) cooling were no significant and the average diameter was close to 1.8 mm. In order to study the influence of the screw speed, 10 rpm was used to obtain the filament (LF10-195). Results showed that an increase in screw speed induced a higher extrudate swelling, as a result of elastic recovery [27], which caused a considerable increase in the average diameter of the filament, being 1.88 ± 0.02 mm. It is important to note that, although the extrusion die had a diameter of 1.7 mm, the obtained diameters in all the previous discussed filaments were above that value. This is a consequence of the conical end of the extrusion die, which increases the swelling effect.

With the aim of achieving a constant diameter along the filaments of 1.7 mm, a new extrusion die was machined without the conical geometry at the output (M code in Table 2). The normal distributions of the diameter of the produced filaments are also shown in Figure 3. Due to the higher length-to-diameter ratio of the machined extrusion die, which increased the difficulty to flow, the normal distribution for AF5-195M was wider than the one for AF5-195. In order to diminish viscosity and facilitate the flow through the extrusion die, the temperature was increased up to 220 °C. This increment in temperature caused the narrowing of the normal distribution (AF5-220M), resulting in a more homogeneous filament with an average diameter of 1.69 ± 0.02 mm, which is adequate for 3D printing. Although 3D printing was feasible with the obtained filament, the output speed was too low and, consequently, the production rate too. For this reason, the speed was increased to 10 rpm (AF10-220M). With this speed, a slightly wider normal distribution was obtained (1.69 ± 0.03 mm), but the characteristics of the filament were considered adequate for 3D printing.

**Figure 4:**
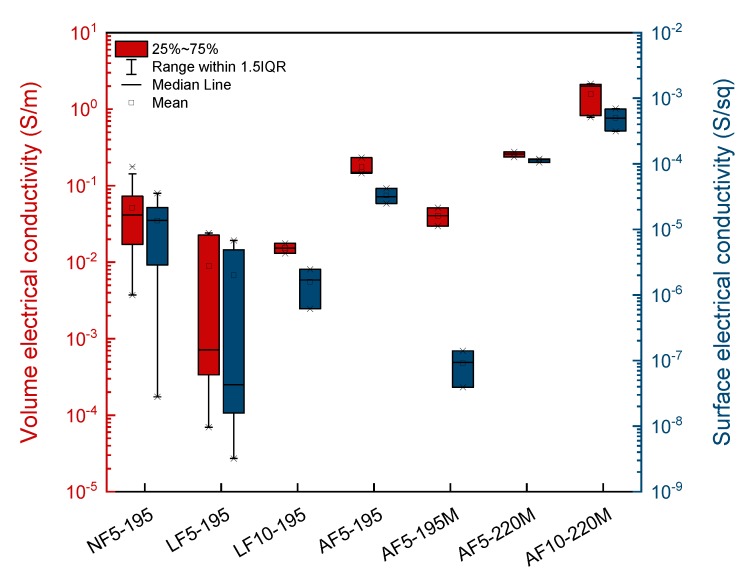
Influence of operational parameters on volume (red) and surface (blue) electrical conductivity (per square) (asterisks represent the outliers).

As a consequence of the variations described above, the volume and surface electrical conductivity of the filament can be also influenced by the extrusion operational parameters. The volume and surface electrical conductivity of the pellets were 5.1 ± 0.5 S/m and (3.3 ± 0.2) · 10^−3^ S/sq, respectively, and Figure 4 shows the electrical conductivity of the obtained filaments. As expected, filaments with narrower diameter normal distributions resulted in more uniform electrical conductivities, and will also lead to more homogenous 3D printed parts.

In all the cases, the surface electrical conductivity of the extruded filaments was more than one order of magnitude lower than the volume electrical conductivity. This fact is due to the reduction in diameter of the filaments, causing a higher surface-to-cross-section ratio. Gonçalves et al. [20] also reported a decrease in electrical conductivity of filaments of CNTs and GNPs reinforced polyether ether ketone (PEEK) associated with the higher surface-to-cross-section ratio of the filaments. Another important parameter influencing electrical conductivity is swelling. As a result of the higher degree of swelling obtained in filaments extruded with the conical output extrusion die, ABS matrix moves to the surface of the filament creating a thin insulating layer, with the consequent decrease in electrical conductivity. Additionally, orientation of the GNPs along the extrusion axis occurs, and this is more effective when using higher extrusion speed rates and higher temperatures, i.e., lower viscosities [28,29,30]. This phenomenon causes an increase in both the volume and surface electrical conductivity of the filaments.

In conclusion, from the results discussed above, the filament combining the highest uniformity in diameter and the highest volume and surface electrical conductivities was AF10-220M. This was the one used to print 3D parts.

### 3.2. Influence of 3D Printing Operational Parameters on the Electrical Conductivity of Printed Parts

As well as extrusion operational parameters strongly influences the volume and surface electrical conductivities of the filaments feeding the 3D printer, operational parameters used in 3D printing also may cause differences in electrical conductivity of printed parts. In order to analyze this dependence, cubic parts using different layer thickness (0.1, 0.2, and 0.3 mm) and width (0.4, 0.6, and 0.8 mm) were manufactured.

**Figure 5:**
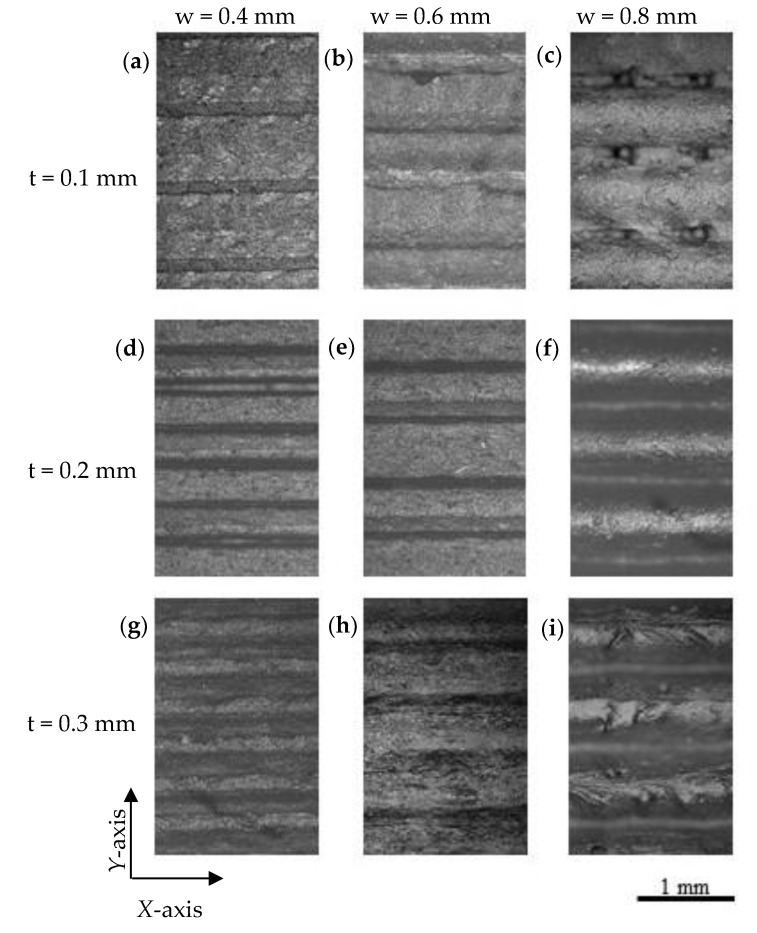
Optical micrographs of the surface of 3D printed parts: (**a**) P1.4, (**b**) P1.6, (**c**) P1.8, (**d**) P2.4, (**e**) P2.6, (**f**) P2.8, (**g**) P3.4, (**h**) P3.6 and (**i**) P3.8 (t = layer thickness, w = extrusion width).

Figure 5 shows representative optical micrographs of the top surface of printed parts. It can be seen that in samples printed using 0.4 mm width (P1.4, P2.4, and P3.4), contact between consecutive lines along *Y*-axis existed for the three layer thicknesses used. When the extrusion width increased up to 0.6 mm (P1.6, P2.6, and P3.6), although there was connectivity along the *Y*-axis, there was a loss in uniformity and some defects could be observed. This fact is attributed to difficulties in flow and high viscosity caused by the presence of the GNPs into the ABS matrix. For a width of 0.8 mm, contact between adjacent deposited filaments was lost, being the space between them 194 ± 24, 106 ± 9, and 82 ± 17 µm for layer thicknesses of 0.1, 0.2, and 0.3 mm, respectively. The space between adjacent extruded lines is due to the low wettability of GNP-based composite materials (static contact angle with water between 91°–131°), which has been reported by other authors to be due to the low surface free energy of the GNPs [31,32,33]. Additionally, as the deposition speed was maintained, the higher the extrusion width or layer thickness, the higher the flow rate and, consequently, the more difficulties to flow through the nozzle tip.

**Figure 6:**
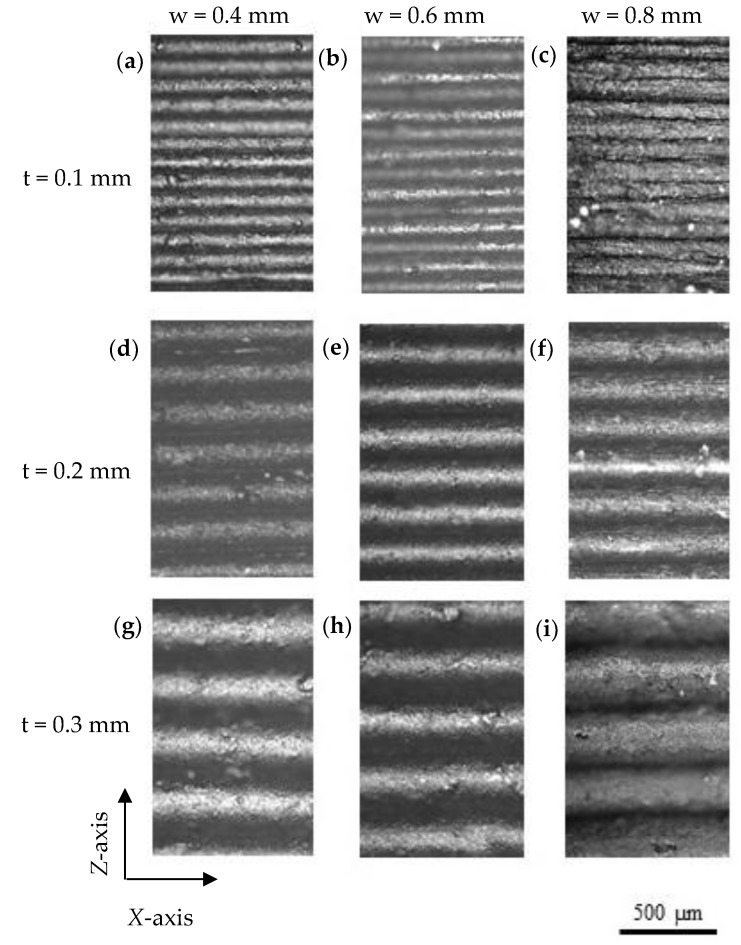
Optical micrographs of the layers thickness of 3D printed parts: (**a**) P1.4, (**b**) P1.6, (**c**) P1.8, (**d**) P2.4, (**e**) P2.6, (**f**) P2.8, (**g**) P3.4, (**h**) P3.6 and (**i**) P3.8 (t = layer thickness, w = extrusion width).

In contrast with variations found in the top surface of samples, the lateral surface (and layer thickness) was homogeneous in printed parts, regardless of the operational parameters. Figure 6 shows representative images of the lateral surface of printed parts. It can be seen that there were no significant differences in thickness depending on the extrusion width and that all the consecutive layers were in contact along the *Z*-axis, ensuring continuity along it.

**Figure 7:**
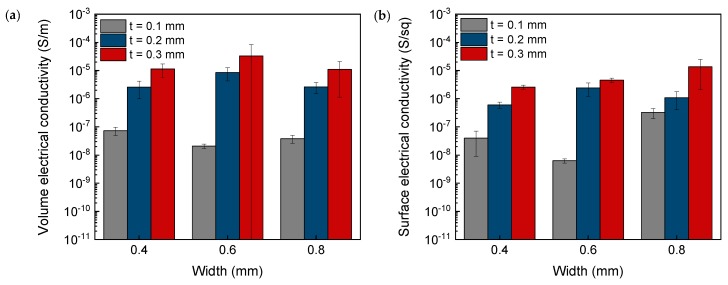
Volume (along *Z*-axis) (**a**) and surface (**b**) electrical conductivity (along *X*-axis) of 3D printed parts.

In order to characterize and analyze the influence of these two parameters in the electrical conductivity of 3D printed parts, Figure 7 shows the volume electrical conductivity along the *Z*-axis (Figure 7a) and the surface electrical conductivity at the top surface along the *X*-axis (Figure 7b), i.e., parallel to the printing lines of the last printed layer. Both the volume and surface electrical conductivity of 3D printed parts were considerably lower compared with the electrical conductivity of the filament used. The reduction of the electrical conductivity in the composite material after 3D printing has been also reported by R. Sanatgar et al. [34] in CNT/polylactic acid (PLA) nanocomposites, who attributed this phenomenon to the diminution of the filament diameter induced by passing through the nozzle tip.

The volume electrical conductivity increased with the printing layer thickness. This increment is due to the fact that the number of layers to achieve the same part height was lower when using higher printing layer thicknesses as well as the higher cross-section area of printed lines for a same width. As a consequence, if the 3D part was considered as electrical resistances in series, constituted by the electrical resistances of each layer ($R_{i}$) and the electrical resistance of the interface between adjacent layers ($R_{i\left(i+1\right)}$), the global electrical resistance ($R_{G}$) was higher as the number of layers increased (Equation (3)).

(3) $$R_{G}=\sum_{1}^{n-1}\left(R_{i}+R_{i\left(i+1\right)}\right)+R_{n},$$

where $R_{n}$ is the electrical resistance of the last printed layer.

Additionally, the volume electrical conductivity along *Z*-axis also decreased when using a lower printing extrusion width, which wasassociated with the diminution of the filament diameter passing through the nozzle tip, already mentioned above. This effect was also appreciated in the surface electrical conductivity. When the cross-section area of printed lines increased, both the volume and surface electrical conductivity along the longitudinal axis grew, resulting in a lower surface electrical resistance. Similar results were obtained by Yang et al. [35], who confirmed that the use of lower layer thickness, i.e., lower cross-section areas, induces an abrupt increase in the surface electrical resistance, which is in agreement with the discussed results.

It is important to clarify that this trend was lost for samples printed with an extrusion width of 0.8 mm, as the volume electrical conductivity diminished. This decrease is caused by the lower contact surface area between subsequent lines through the *Z*-axis, already mentioned, which increases the electrical resistance at the interface of adjacent layers. Other deviation of the trend was also observed in P1.6 samples, which also showed lower volume and surface electrical conductivities due to the presence of discontinuities (already discussed in the previous section).

Although the electrical conductivity obtained in 3D printed parts was lower than that of the filament prior to printing, the values were in the order of other reported parts manufactured by extrusion-based AM, as can be corroborated from data included in Table 4. It has been demonstrated that the electrical conductivity of parts obtained by extrusion-based AM is reduced at least one order of magnitude with respect to conventional methods as compression molding (CM) [36].

**Figure 8:**
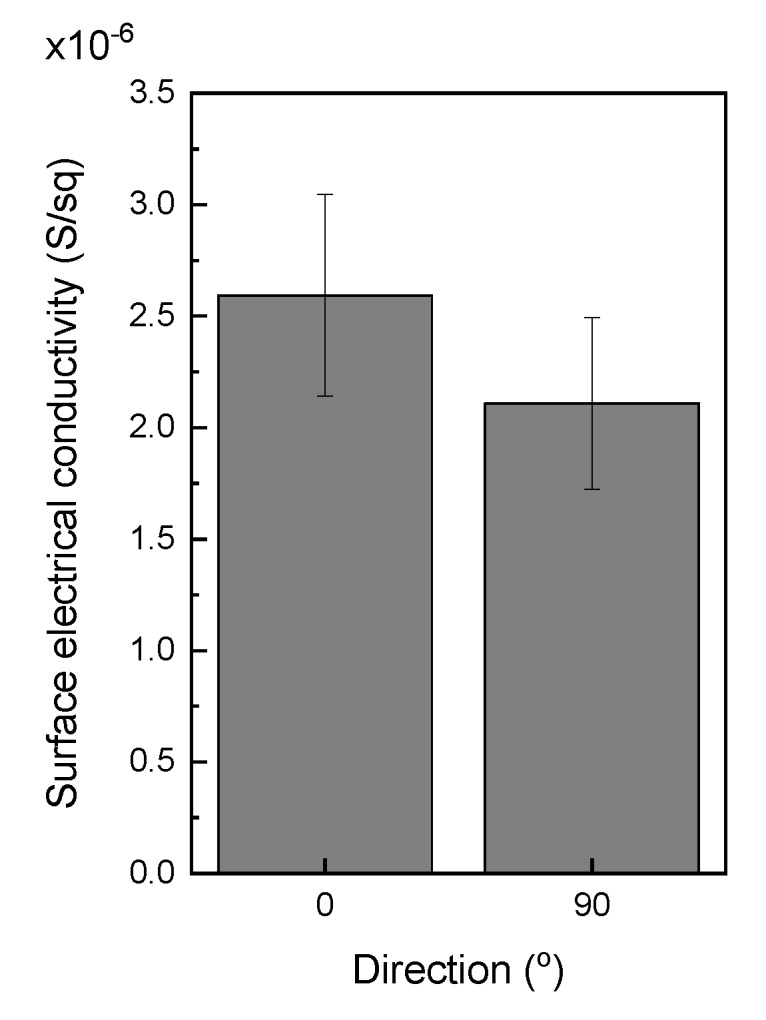
Surface electrical conductivity along *X*-axis and *Y*-axis of P3.4 printed parts.

Another issue to consider is the anisotropy of the 3D printed parts, as there is preferential orientation of the electrically conductive nanoparticles when the composite pass through the nozzle tip. D. Zhang et al. already reported the preferential orientation that experienced GNPs during 3D printing [41]. This statement is also supported by Goh et al. [42], who corroborated the alignment of CNTs along the extrusion direction. In order to study the anisotropy of the 3D printed parts, the surface electrical conductivity was measured along the *X*-axis (0°) and *Y*-axis (90°). The results are shown in Figure 8. The electrical conductivity along the *Y*-axis, i.e., perpendicular to the printed lines, was lower than that along the *X*-axis. This anisotropy was attributed to the preferential orientation of the GNPs, already mentioned above, but also to the directionality of the manufacturing process. Parallel to the printed lines (*X*-axis), the system acted as resistances in parallel while perpendicular to them (*Y*-axis), the system geometry is similar to that explained for volume electrical conductivity through *Z*-axis, as resistances in series.

### 3.3. Influence of Surface Post Processing on the Electrical Conductivity of Printed Parts

**Figure 9:**
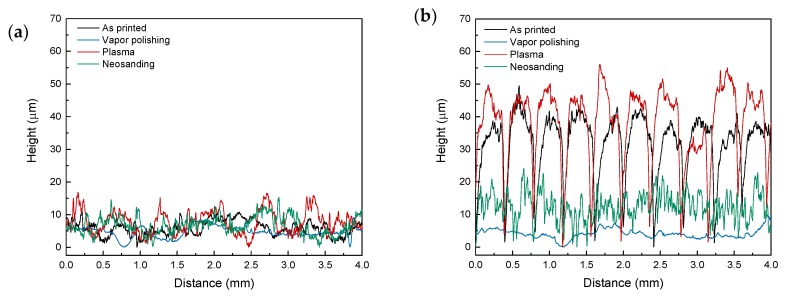
Surface profiles of as printed and surface post processed parts: (**a**) *X*- and (**b**) *Y*- profiles.

Post processing of the surface of 3D printed parts has been analyzed in order to enhance the roughness and surface electrical conductivity. Vapor polishing, plasma treatment and neosanding were used to modify the surface properties. Figure 9 shows the surface profiles to elucidate geometrical changes induced by post processing. Along *X*-axis (Figure 9a), i.e., 0°, significant variations were not observed, except for vapor polishing, that caused a reduction of roughness. Along *Y*-axis (Figure 9b), i.e., 90°, while vapor polishing and neosanding caused a reduction of roughness (more significant in the case of vapor polishing), plasma did not make appreciable changes.

**Figure 10:**
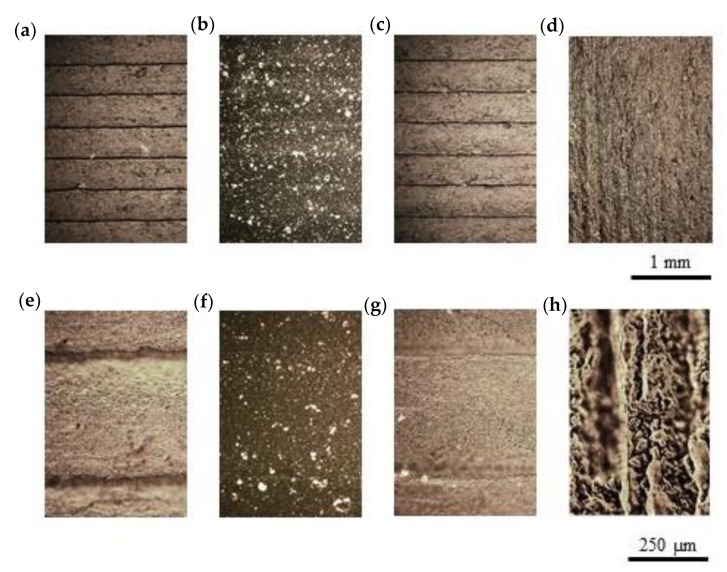
Optical micrographs of surface post processed parts: (**a**,**e**) as printed, (**b**,**f**) vapor polishing, (**c**,**g**) plasma and (**d**,**h**) neosanding.

These geometrical features can be appreciated in Figure 10, which shows representative optical micrographs of the top surface of treated parts at different magnifications. Related to the as printed part (Figure 10a,e), vapor polishing surfaces (Figure 10b,f) showed a continuous surface with slight differentiation of the printed lines [43]. Another representative issue of polishing, due to the low roughness, is that leads to transparency, thus making evident the presence of GNPs into the ABS matrix. Plasma treated parts (Figure 10c,g) did not produce significant changes. In contrast, neosanding (Figure 10d,h) changed the periodicity of the roughness and the preferential orientation, which was now parallel to the post processing direction (*Y*-axis).

**Figure 11:**
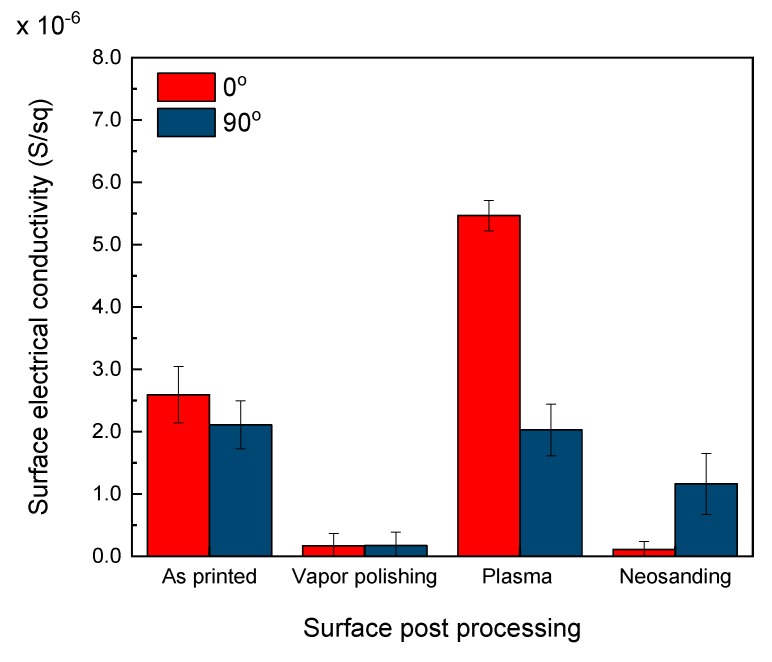
Electrical conductivity of surface post processed parts: as printed, vapor polishing, plasma and neosanding.

With the aim of analyzing the influence of post processing in surface electrical conductivity of 3D printed parts, this was measured along *X*-axis (0°) and *Y*-axis (90°). Results are shown in Figure 11. Vapor polishing caused a reduction of the electrical conductivity of near one order of magnitude with respect to the as printed part. The reason of the decrease is that during vapor polishing, acetone dissolved ABS, allowing the polymer to flow [44,45] and, consequently, a thin insulating layer is formed on the top, increasing the surface electrical resistance.

On the contrary, plasma treated parts showed a surface electrical conductivity nearly three times higher along *X*-axis (0°). In contrast, along *Y*-axis (90°) there was no variation because of the electrical resistance of the interface between adjacent deposited lines. This increase is produced because the surface becomes more hydrophilic due to the plasma functionalization [24], which increases the polar surface energy because of the induction of polar functional groups [46]. Neosanding also caused significant differences in the surface electrical properties. The surface electrical conductivity along *X*-and *Y*-axis was reduced, but it was higher along *Y*-axis, which coincided with the parallel direction to the movement of the nozzle tip. This post processing induced the realignment of the GNPs of the surface and changes the orientation of the preferential direction of the roughness in the direction of the process, reversing the surface electrical conductivity tendencies.

Although neosanding was demonstrated to be a process suitable to tune the surface properties of 3D printed parts, the reproducibility is currently a challenge. Samples processed with the same neosanding parameters in different batches showed surface electrical conductivities in the range of 10^−7^–10^−5^ S/sq. For this reason, precision is a key factor to have reproducible surface processing procedures.

## 4. Conclusions

The key role of the manufacturing parameters of extrusion (refrigeration type, screw speed, extrusion temperature and output shape of the die) and 3D printing (layer thickness and extrusion width) to obtain electrically conductive filaments and 3D printed parts, respectively, was demonstrated.

The results have shown that an increase in screw speed, as well as lower land lengths, induces higher extrudate swelling, with the consequent reduction of the electrical conductivity (compared with the value obtained for the pellets). Additionally, filaments with lower diameter values, which result in a higher surface-to-cross-section ration, have considerably lower electrical conductivities. Homogeneous refrigeration has also been demonstrated to be essential to obtain stability in the diameter along the extruded filament and, therefore, in the surface and electrical conductivity. These factors tune the values of the volume and surface electrical conductivity between 10^−4^–10^0^ S/m and 10^−8^–10^−3^ S/sq, respectively.

The operational parameters in 3D printing have also shown to be crucial to maximize the electrical conductivity. In the absence of defects, the volume electrical conductivity of 3D printed parts is enhanced by increasing the printing layer thickness and extrusion width. This improvement is caused by two effects. On the one hand, the number of layers to achieve the same part height is lower when using higher printing layer thicknesses, thus leading to reduced electrical resistance. On the other hand, the higher the layer thickness and extrusion width are, the greater the cross-section area of the printed lines, resulting in a lower electrical resistance.

The effect of different post processing of 3D printed parts in morphology and surface electrical conductivity was also analyzed. While acetone vapor polishing induced a diminution in surface electrical conductivity close to one order of magnitude; enhancement of the electrical conductivity along the *X*-axis was obtained for plasma treated parts. Neosanding post processed samples showed surface electrical conductivities in the range of 10^−7^–10^−5^ S/sq. This variability demonstrated that precision is a key factor to have reproducible results in this novel technique.

## Figures and Tables

**Table 1:** Acrylonitrile butadiene styrene ABS properties.

Density (ISO 1183/B)	1.05 g/cm^3^
Melt Mass-Flow Rate (220 °C) (ISO 1133)	15 g/10 min
Tensile Modulus (ISO 527-2)	2280 MPa
Tensile Stress (ISO 527-2/50)	45 MPa
Tensile Strain (ISO 527-2/50)	2.5%
Flexural Modulus (ISO 178)	2300 MPa
Flexural Stress (ISO 178)	68 MPa
Heat Deflection Temperature (1.84 MPa, annealed)	100 °C

**Table 2:** Operational parameters for filament extrusion.

Filament Code	Refrigeration	Screw Speed (rpm)	Temperature (°C)	Output
NF5-195	Air cooling	5	195	Conical
LF5-195	Linear fan	5	195	Conical
LF10-195	Linear fan	10	195	Conical
AF5-195	Annular fan	5	195	Conical
AF5-195M	Annular fan	5	195	Cylindrical
AF5-220M	Annular fan	5	220	Cylindrical
AF10-220M	Annular fan	10	220	Cylindrical

**Table 3:** Operational parameters for 3D printed parts.

Part ID	Layer Thickness (mm)	Extrusion Width (mm)	Orientation (°)
P1.4	0.1	0.4	0/90
P1.6		0.6	
P1.8		0.8	
P2.4	0.2	0.4	
P2.6		0.6	
P2.8		0.8	
P3.4	0.3	0.4	
P3.6		0.6	
P3.8		0.8	

**Table 4:** Electrical conductivity of ABS-matrix nanocomposites.

Material	Process	Dimensions (mm^3^)	Percolation Threshold (wt %)	Content (wt %)	Electrical Conductivity (S/m)	Ref.
Pure ABS	-	-	-	-	10^−15^	[37]
ABS + GNPs	FDM	10 × 10 × 10		15	10^−8^–10^−5^	Current work
ABS + GNPs	CM ^1^	45 × 45 × 2	-	6	10^−12^	[36]
	FDM ^2^				10^−13^	
ABS + CNTs	CM ^1^		-		10^−1^	
	FDM ^2^				10^−2^	
ABS + CNTs	FDM ^2^	50 × 6 × 1.2	1	6	10^0^–10^1^	[18]
ABS + rGO	Hot press	Ø 5 cm	0.3	1.3	10^−4^	[38]
				2	10^−3^	
				3	10^−2^	
ABS + GNPs	CM ^1^	64 × 64 × 1.2	8–12	20	10^−2^	[39]
ABS + CNTs			2	8	10^2^	
ABS + GNPs	Hot press	64 × 64 × 1.2	-	8	10^−12^	[40]

^1^ Compression molding. ^2^ Fused Deposition Modeling.
